# Validity and screening capacity of the FCR-1r for fear of cancer recurrence in long-term colorectal cancer survivors

**DOI:** 10.1007/s00520-023-08159-7

**Published:** 2023-11-11

**Authors:** Johanne Dam Lyhne, Allan “Ben” Smith, Signe Timm, Sébastien Simard, Lars Henrik Jensen, Lisbeth Frostholm, Per Fink

**Affiliations:** 1 Department of Clinical Oncology, University Hospital of Southern Denmark, Beriderbakken 4, 7100 Vejle, Denmark; 2 South West Sydney Clinical Campuses, Faculty of Medicine and Health, University of New South Wales (UNSW Sydney), Liverpool, Australia; 3 Ingham Institute for Applied Medical Research, Liverpool Hospital, Locked Bag 7103, Liverpool BC, NSW 1871 Australia; 4 Université du Québec À Chicoutimi (UQAC), Health Sciences Department, 555, Boul. de L’Université, Chicoutimi (Qc), Canada; 5 Research Clinic for Functional Disorders and Psychosomatics, Aarhus University Hospital, Palle Juul-Jensens Boulevard 99, 8200 Aarhus N, Denmark; 6 Department of Clinical Medicine, Aarhus University, Nordre Ringgade 1, 8000 Aarhus C, Denmark

**Keywords:** Fear of cancer recurrence, Validation, Screening, Colorectal cancer, Survivorship care, Psycho-oncology

## Abstract

**Purpose:**

Existing fear of cancer recurrence (FCR) screening measures is being shortened to facilitate clinical use. This study aimed to evaluate the validity and screening capacity of a single-item FCR screening measure (FCR-1r) in long-term colorectal cancer (CRC) survivors with no recurrence and assess whether it performs as well in older as in younger survivors.

**Methods:**

All Danish CRC survivors above 18, diagnosed and treated with curative intent between 2014 and 2018, were located through a national patient registry. A questionnaire including the FCR-1r, which measures FCR on a 0–10 visual analog scale, alongside the validated Fear of Cancer Recurrence Inventory Short Form (FCRI-SF) as a reference standard was distributed between November 2021 and May 2023. Screening capacity and cut-offs were evaluated with a receiver-operating characteristic analysis (ROC) in older (≥ 65 years) compared to younger (< 65 years) CRC survivors. Hypotheses regarding associations with other psychological variables were tested as indicators of convergent and divergent validity.

**Results:**

Of the CRC survivors, 2,128/4,483 (47.5%) responded; 1,654 (36.9%) questionnaires were eligible for analyses (median age 76 (range 38–98), 47% female). Of the responders, 85.2% were aged ≥ 65. Ninety-two participants (5.6%) reported FCRI-SF scores ≥ 22 indicating clinically significant FCR. A FCR-1r cut-off ≥ 5/10 had 93.5% sensitivity and 80.4% specificity for detecting clinically significant FCR (AUC = 0.93, 95% CI 0.91–0.94) in the overall sample. The discrimination ability was significantly better in older (AUC = 0.93, 95% CI 0.91–0.95) compared to younger (0.87, 95% (0.82–0.92), *p* = 0.04) CRC survivors. The FCR-1r demonstrated concurrent validity against the FCRI-SF (*r* = 0.71, *p* < 0.0001) and convergent validity against the short-versions of the Symptom Checklist-90-*R* subscales for anxiety (*r* = 0.38, *p* < 0.0001), depression (*r* = 0.27, *p* < 0.0001), and emotional distress (*r* = 0.37, *p* < 0.0001). The FCR-1r correlated weakly with employment status (*r* =  − 0.09, *p* < 0.0001) and not with marital status (*r* = 0.01, *p* = 0.66) indicating divergent validity.

**Conclusions:**

The FCR-1r is a valid tool for FCR screening in CRC survivors with excellent ability to discriminate between clinical and non-clinical FCR, particularly in older CRC survivors.

**Supplementary Information:**

The online version contains supplementary material available at 10.1007/s00520-023-08159-7.

## Introduction

As more people are living longer with and after cancer, there is a growing proportion of older survivors, raising the importance of conducting research with these populations. While many long-term cancer survivors do not face the acute stressors of treatment and follow-up, they continuously deal with the uncertainties of survivorship, including potential recurrence. Fear of cancer recurrence (FCR), defined as *“fear, worry or concern that cancer will come back or progress”* [1] is a top health-related concern for almost all cancer survivors [2], and help managing FCR is one of the most frequently reported unmet supportive care needs [3–5].

Identifying survivors with clinically significant levels of FCR is important, as it can greatly affect quality of life and may persist for many years without treatment [6, 7]. On average, older long-term cancer survivors report less FCR compared to younger survivors [8], independent of cancer type [7, 9, 10], but high levels of FCR are reported in a small but significant group of older (mean age 77.6 years), long-term (average time since diagnosis 9.5 years) cancer survivors (15.9% according to a Cancer Worry Scale cut-off ≥ 14) [7].

Psychosocial screening of older cancer survivors for multiple psychosocial issues with numerous long questionnaires is problematic, as age and anticancer treatment may together and separately affect cognitive function [11]. Besides this, screening requires administration, scoring, and interpretation by a healthcare provider, all of which may be complicated by lengthy questionnaires.

Efforts have been made to create short, simple, and valid screening items. The single-item FCR measure (FCR-1) [12] with the verbal formulation *“*On a scale from 0 to 100, what is your subjective level of fear of cancer recurrence at this time?” was developed for use in group sessions aimed at reducing FCR. The FCR-1 has been psychometrically tested [12] and then revised and validated in written form, FCR-1r [13].

In both studies, validation was performed in relatively small samples (FCR-1; *N* = 69 and FCR-1r; *n* = 107). The psychometric properties of the FCR-1 were evaluated only in women with breast and gynecological cancer with a mean age of 55, and the FCR-1r was evaluated in a group of patients with mixed cancers where 15% had experienced a cancer recurrence. There is a need to further validate the FCR-1r in additional populations with different types of cancer and at various stages of survivorship to extend generalizability.

The primary objective of this cross-sectional study was to examine the psychometric properties of the FCR-1r, by examining its concurrent, convergent, and divergent validity in a sample of long-term colorectal cancer (CRC) survivors and to investigate the screening performance of the FCR-1r in older versus younger CRC survivors. Results can be used to support the integration of a short and simple FCR screening item into routine psychosocial screening for CRC survivors.

## Materials and methods

### Design

This cross-sectional study followed the COSMIN Study Design checklist for Patient-reported outcome measurement instruments [14]. The data presented is part of a national study of the prevalence of psychosocial late effects in long-term (defined as ≥ 3 years since cancer diagnosis with no recurrence and no residual disease) CRC survivors in Denmark, which subsequently will be used for recruitment to a randomized controlled trial (RCT) of a therapist-guided online FCR intervention (see published protocol) [15].

### Sample and recruitment procedures

All CRC survivors in Denmark are included considering the following: (a) above 18 years; (b) diagnosed with colorectal cancer between 2014 and 2018; and (c) treated with curative intent were invited to participate in the population-based study of psychosocial late effects after CRC from which the sub-sample for this analysis was derived. CRC survivors were located through the Danish Clinical Quality Program–National Clinical Registries (RKKP) which also provided additional data (see later). For this study, data gathered between November 2021 and May 2023 was used.

The survey from “Vejle Hospital” was distributed electronically to a personal secure electronic mailbox (e-Boks) when possible, or as a paper-and-pencil survey with a cover letter presenting the study aim when patients had opted out of receiving electronic mail from the authorities. The survey included an item seeking consent for anonymous responses to be used for research. It was not possible to follow-up non-respondents. Electronic surveys were sent out in a quantity comparable to paper-and-pencil surveys (a sub-sample of 20%).

Returned questionnaires were excluded in cases with a missing social security number (for identification) or if the terms “dementia,” “blindness,” “too ill,” “cancer recurrence,” or “dead” were noted on the questionnaire or communicated to the primary investigator by telephone. In certain instances, survivors indicated “*no memory of cancer*,” “*do not want to participate*,” or “*do not give consent for answers being used for research purposes*,” leading to their exclusion. Participants who responded “0” to every FCRI-SF item, seemingly missing the reversed wording of item 9 “*I believe that I am cured and that the cancer will not come back*” were excluded, as this was interpreted as inattention or lack of motivation [16]. Paper-and-pencil questionnaire responses were manually entered into a REDCap database, with 10% double data entry for quality assurance. Responses received through e-Boks were directly uploaded into REDCap.

### Data collection

The survey included clinical and demographic questions, and a battery of validated questionnaires, two of which (the Fear of Cancer Recurrence Inventory Short Form (FCRI-SF) [17] and the Symptom Checklist-90-R (SCL) [18]) were validated in Danish. The other two (the FCR-1r and the Global Quality of Life (QoL)) were translated and field-tested during this study. The translation was a collaborative effort between a trilingual research administrator and the primary investigator. It was further validated through field testing, involving an age-matched group of three ordinary people and three cancer survivors. The translation remained unchanged after the field-testing process.

The FCRI-SF [19] is the most widely validated and used FCR screening tool in a research context, but its 9-item length makes it impractical for use in routine survivorship care. Item response categories are on a 5-point Likert-like scale ranging from (0) not at all/never to (4) a great deal/all the time. Scale scores range from 0 to 36 with higher scores indicating greater FCR severity. The criterion validity of the FCRI-SF has been demonstrated in a population (*n* = 60, mean age 60) of short-term (mean 1.9 years) mixed cancer survivors with a face-to-face FCR interview as the gold standard [20]. Originally, two cut-off scores were validated (13/36 to indicate any level of FCR and 16/36 to indicate potential severe FCR). In this study, the more recent FCRI-SF cut-off of 22/36 was used due to its higher sensitivity (90%) and specificity (83.3%) for identifying clinical cases of FCR according to clinical interviews [21].

The FCR-1r [13] is the adaptation of the verbally administered FCR-1 [12] to a written form compatible with the Edmonton Symptom Assessment System (ESAS) [22], developed for use in routine clinical care. Respondents are asked to “*Please circle the number that best describes how you feel NOW* on a visual analog scale from *0* = *No FCR to 10* = *Worst possible FCR (FCR* = *fear that your cancer will come back or get worse).”* In the study by Smith et al., the FCR-1r was strongly correlated to FCRI-SF (*r* = 0.83, *p* < 0.0001). As only survivors with no history of recurrence were included in the present study, “*or get worse*” was deleted.

The short version of the Symptom Checklist-90-R (SCL) [23] was used along with the anxiety subscale (SCL-anx) [24], depression subscale (SCL-dep), and general distress subscale (SCL-distress) [18] to measure symptoms of anxiety, depression, and emotional distress, respectively, during the previous four weeks on a 5-point Likert like scale rating from (0) not at all to (4) extremely. For each subscale, a sum score is calculated with higher scores indicating more severe symptoms. The SCL-anx, SCL-dep, and SCL-distress subscales use 4, 6, and 8 items respectively, resulting in sum scores ranging from 0 to 16, 24, and 32 respectively. The SCL has undergone psychometric testing in a primary care cohort of 701 patients as a part of the Common Mental Disorder Questionnaire (CMDQ) [25, 26]. The diagnostic performance for scores above/below cut-offs to correctly classify those with ICD-10 diagnoses according to psychiatric research interview (SCAN) was excellent. The area under the curve (AUC) for SCL-dep was 0.88 (95% CI 0.84–0.91) and for SCL-anx 0.87 (95% CI 0.82–0.92). The SCL distress also performed well, with an AUC of 0.78 (95% CI 0.71–0.85) [26].

Global QoL was rated using a single visual analog scale (VAS) inspired by the EQ-5D-5L [27]. The word “health” was changed into “quality of life,” and participants were asked to self-rate their overall quality of life TODAY on a VAS from 0 to 100 with 0 being *“The worst quality of life you can imagine”* and 100 being *“The best quality of life you can imagine.”* A comparable single-item QoL VAS showed good validity in a population of 83 patients with esophageal adenocarcinoma compared to the Medical Outcomes Study Short Form-20 and the Rotterdam Symptom Check-List [28].

Clinical and demographic questions referred to time since follow-up appointment and time to the next follow-up if not finished, chemotherapy received (yes/no), radiotherapy received (yes/no), marital status, employment status, education (any or none) citizenship (Danish or other), and children (yes/no) were also included in the survey.

### Registry data

Information on participant characteristics (age and sex), clinical characteristics (cancer type (colon or rectum)), and diagnosis date and tumor stage (metastatic disease (yes/no)) were retrieved directly from the Danish Clinical Quality Program – National Clinical Registries (RKKP) along with WHO performance status at time of operation.

### Statistical analysis

Analyses were performed using STATA17 (StataCorp, College Station, TX, USA). Descriptive statistics were used to summarize demographics and clinical characteristics. According to our informal criterion, an optimal rate of missing responses is less than 3% per item. If more than 15% were missing, this was considered not acceptable. In-between rates of missing data were imputed [29] with both mean imputation (imputation of participant’s own mean value of remaining answers) and multiple imputation (using mvn-algorithm and gender as auxiliary variable to impute 20 iterations of datasets) to explore differences between imputation approaches and complete case analyses. Missingness was assumed to be missing at random (MAR) [30], based on explorative analysis on a comparable population of CRC survivors (unpublished data).

#### Concurrent validity

For concurrent validity, associations between FCR-1r and FCRI-SF scores were assessed using Spearman’s Rho.

Construct validity was examined by testing a priori hypotheses on direction of associations between scores on the FCR-1r and relevant psychological measures. Spearman’s Rho was used as the distributions of the variables were not normal. Associated confidence intervals were calculated by bootstrapping. Difference in gender was analyzed by the Wilcoxon rank-sum test. Correlations were considered strong if > 0.70, moderate if 0.30–0.69 and low if < 0.30 [31].

Built upon rational and theoretical choices as well as previous research [2, 6, 8, 10, 32, 33], we hypothesized that the FCR-1r—similar to the FCRI-SF—would show the following:

#### Convergent validity

1. A significant positive correlation between SCL-anx, SCL-dep, and SCL-distress.
2. A weaker correlation with the SCL-dep than with the SCL-anx.
3. A positive correlation between younger age and female gender, both of which are associated with greater FCR.
4. A negative correlation with Global QoL.

#### Divergent validity

5. Correlations of − 0.10 to 0.10 with marital status and employment status, i.e., no or only very small association between FCR and these demographic factors, as generally noted in the literature [34]

### Screening capacity

For the two age groups and for the overall sample, receiver operating characteristic (ROC) curves evaluated the ability of the FCR-1r to discriminate between:

- Any level of FCR (FCRI-SF ≥ 13) and no FCR
- Potential severe FCR (FCRI-SF ≥ 16) and no potential severe FCR
- Clinical (FCRI-SF ≥ 22) and non-clinical levels of FCR

Discrimination ability of the FCR-1r for all three cut-offs will be presented (partly in supplementary material) as applicability is dependent on the screening context.

A suggestion of an optimal cut-off score on the FCR-1r was determined based on evaluation of sensitivity, specificity, and negative (NPV) and positive predictive value (PPV), which were calculated for FCR-1r scores 0–10.

## Results

**Fig. 1:**
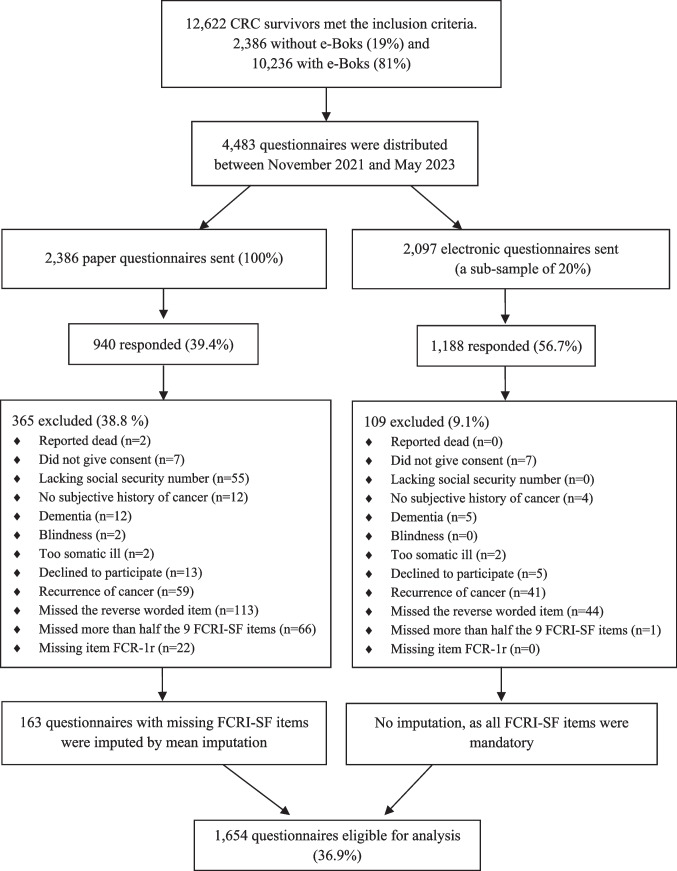
Participant flow chart

Of the 4,483 CRC survivors, 2,128 (47.5%) returned the survey. 474 responses were excluded during data verification (Fig. 1). Among these were 157 cases with a response pattern of “0” to every FCRI-SF item. 1,654 participants had complete data on both FCR-1r and FCRI-SF after imputation, and were included in the analyses (median age 76 years (range 38–98), 47% females). (See Table 1 for socio-demographic and clinical characteristics for the two age groups (< 65 years and ≥ 65 years) and for the overall sample.) Mean FCR-1r score was 2.6/10 and mean FCRI-SF score was 10.1/36. Non-responders and those who were excluded were most prominent in paper surveys, but CRC survivors willing and eligible to respond were no different from CRC survivors not willing to respond regarding clinical and demographic characteristics (see Table 1a (*paper* population) and Table 1b (*e-Boks* population) in supplementary material). No unexpected differences were seen between paper and e-Boks survey respondents. E-boks responders tended to be younger and reported higher FCR levels. E-Boks responders were more likely to have rectal cancer, and a higher percentage were married (as opposed to widowed) and were still employed (see Table 2 in supplementary material).

**Table 1:** Demographic and clinical characteristics for respondents under age 65, 65 or above and for the total sample. Numbers in parentheses are percentages unless otherwise specified

	*Under age 65, N* = *245*	*65 or above, N* = *1,409*	*Total* = *1,654*
Age in years, median, mean, range (sd)	60.1, 59, 38–64.9 (4.9)	77.6, 77.8, 65–98 (6.9)	76, 75, 38–98 (9.4)
Age categories			
< 45	5 (2%)		5 (0%)
45–55	35 (14%)		35 (2%)
55–65	205 (84%)		205 (12%)
65–75		499 (35%)	499 (30%)
75–85		693 (49%)	693 (42%)
85–95		207 (15%)	207 (13%)
> 95		10 (1%)	10 (0%)
Gender, female	125 (51%)	657 (47%)	782 (47%)
Cancer type			
Colon	155 (62%)	974 (69%)	1,129 (68%)
Rectum	90 (37%)	435 (31%)	525 (32%)
Mean FCR-1r score (sd)	3.5 (2.7)	2.4 (2.6)	2.6 (2.6)
Mean FCRI-SF score (sd)	12.8 (6.7)	9.7 (6.5)	10.1 (6.6)
Years since diagnosis, mean, range (sd)	6.5, 2.9–9.2 (1.4)	6.2, 2.9–9.2 (1.5)	6.3, 2.9–9.2 (1.5)
Time since the last follow-up			
> 1 year	151 (62%)	963 (68%)	1,114 (67%)
3–12 months	68 (28%)	311 (22%)	379 (23%)
1 week–3 months	24 (10%)	94 (7%)	118 (7%)
< 1 week	1 (0%)	16 (1%)	17 (1%)
Missing	1 (0%)	25 (2%)	26 (2%)
Time to next follow-up			
< 1 week	2 (1%)	8 (1%)	10 (1%)
1 week–3 months	12 (5%)	97 (7%)	109 (7%)
3 months–12 months	48 (20%)	139 (10%)	187 (11%)
> 1 year	89 (36%)	280 (20%)	369 (22%)
Cancer control is ended	93 (38%)	845 (60%)	938 (57%)
Missing	1 (0%)	40 (3%)	41 (2%)
Chemotherapy received	125 (51%)	416 (30%)	546 (33%)
Radiotherapy received	24 (10%)	101 (7%)	125 (76%)
Metastatic disease at the time of diagnosis			
No	228 (93%)	1,336 (95%)	1,564 (95%)
Yes	15 (6%)	61 (4%)	76 (5%)
Missing	2 (1%)	12 (1%)	14 (1%)
WHO performance status, mean (sd)	0.1 (0.4)	0.3 (0.6)	0.3 (0.5)
Marital status			
Married	161 (66%)	794 (56%)	955 (58%)
Not married	37 (15%)	93 (7%)	130 (8%)
Divorced/separated	27 (11%)	106 (8%)	133 (8%)
Widowed	5 (2%)	345 (24%)	350 (21%)
Living together	15 (6%)	65 (5%)	80 (5%)
Missing	0	6 (0%)	6 (0%)
Employment status			
Employed	175 (71%)	108 (8%)	283 (17%)
Has been employed	61 (25%)	1,234 (88%)	1,295 (78%)
Has never been employed	4 (2%)	44 (3%)	48 (3%)
Missing	5 (2%)	23 (2%)	28 (2%)
Education, any level, yes	189 (77%)	1,030 (73%)	1,219 (74%)
Citizenship, Danish	236 (96%)	1,354 (96%)	1,590 (95%)
Children, yes	207 (84%)	1,214 (86%)	1,421 (86%)

### Imputation

Only FCRI-SF data were imputed. In 163 (27%) *paper questionnaires,* missing values were observed in up to 4/9 FCRI-SF items (see Table 3 in supplementary material). No difference was found between results using different imputation styles (see Table 4 in supplementary material), and mean imputation was used for further statistics. After imputation, 34 cases (5.7%) reported FCRI-SF score above the cut-off of ≥ 22, but three of these cases had missing data on FCR-1r leaving 31 cases. Scores on SCL subscales were calculated only in complete cases. A unimodal distribution was observed for both FCR-1r and FCRI-SF scores with 40% and 9% reporting 0.

#### Concurrent validity

A strong positive correlation between FCR-1r and FCRI-SF score was observed (*r* = 0.71, *p* < 0.0001). The strongest correlation (*r* = 0.72, *p* < 0.0001) was found between FCR-1r and FCRI-SF item 1 (*I am worried or anxious about the possibility of cancer recurrence),* and the weakest correlation (*r* = 0.18, *p* < 0.0001) with FCRI-SF item 5 (*I believe that I am cured and that the cancer will not come back*). Correlations of FCRI-SF items 2–4 and 6–9 were all between 0.45 and 0.68 (see Table 5 in supplementary material).

### Hypothesis testing

#### Convergent validity

Moderate correlations were observed between the FCR-1r and SCL-anx, SCL-dep, and SCL-distress (see Table 2). The correlation with SCL-dep was weaker than with SCL-anx. Small but significant correlations were observed between FCR-1r scores and lower age and QoL. The magnitude of correlations was similar for the FCRI-SF. Females reported higher FCR-1r and FCRI-SF scores than males (see Table 6 in supplementary material).

**Table 2:** Correlations between the FCR-1r and the FCRI-SF score and psychological and related and unrelated demographic measures

	FCR-1r Spearman’s correlation	*p*	FCRI-SF score Spearman’s correlation	*p*
FCRI-SF	0.71, CI (0.68–0.74)	*p* < 0.0001		
Anxiety (SCL-anx)	0.38, CI (0.34–0.43)	*p* < 0.0001	0.43, CI (0.39–0.47)	*p* < 0.0001
Depression (SCL-dep)	0.27, CI (0.22–0.32)	*p* < 0.0001	0.34, CI (0.29–0.38)	*p* < 0.0001
Emotional distress (SCL-distress)	0.37, CI (0.32–0.41)	*p* < 0.0001	0.42, CI (0.38–0.46)	*p* < 0.0001
Quality of life (Global QoL)	− 0.24, CI (− 0.29– − 0.20)	*p* < 0.0001	− 0.25, CI (− 0.30– − 0.20)	*p* < 0.0001
Age	− 0.22, CI (− 0.26– − 0.17)	*p* < 0.0001	− 0.24, CI (− 0.28– − 0.19)	*p* < 0.0001
Marital status	− 0.01 CI (− 0.06–0.04)	*p* = 0.66	0.02 CI (− 0.03–0.07)	*p* = 0.41
Employment status	− 0.09 CI (− 0.14–0.05)	*p* < 0.0001	− 0.11 CI (− 0.16– − 0.06)	p < 0.0001

#### Divergent validity

A very small association between FCR and demographic factors was observed (see Table 2).

### ROC analyses

Using the FCRI-SF cut-off of 22 or greater, there were 92 positive cases of likely clinically significant FCR in the total sample. These cases were distributed with 26 (10.6%) in the “young” group, and 66 (4.7%) in the “old” group.

**Fig. 2:**
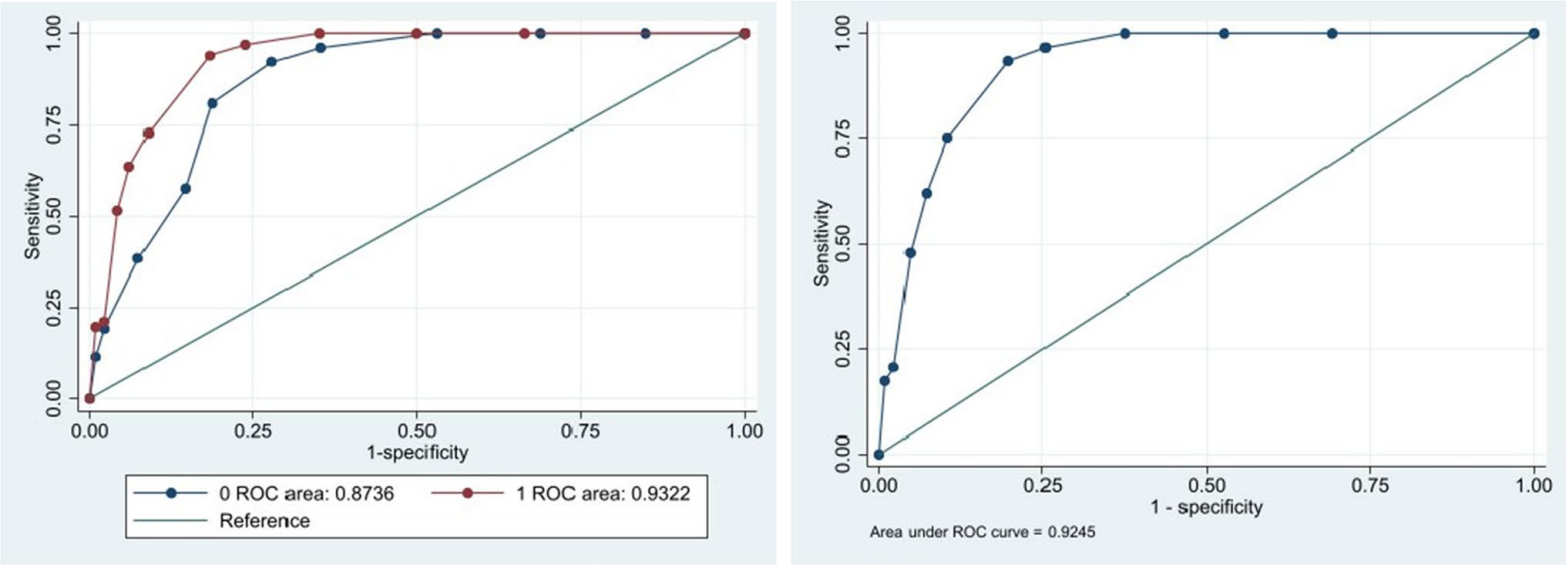
Receiver operating characteristic (ROC) curve evaluating the ability of the FCR-1r to discriminate between clinical and non-clinical FCR (Cut-off ≥ 22) in two age groups (left, blue < 65 years, red ≥ 65 years) and in the total sample of long-term colorectal cancer survivors (right)

The AUC for the two age groups separately and for the total sample are presented in Fig. 2 The AUC for the total sample was 0.92 (95% CI 0.91–0.94, *p* < 0.0000) for the FCR-1r indicating excellent classification ability.

The AUCs were significantly different between the two age groups (< 65 years = 0.87 CI (0.82–0.92) versus ≥ 65 years = 0.93 CI (0.91–0.95), *p* = 0.04), indicating a better screening performance of the FCR-1r in the older group.

A cut-off score of ≥ 5/10 on the FCR-1r was deemed best suited to screen for likely clinically significant FCR with a sensitivity of 93.5% and a specificity of 80.3% (see Table 3). At this cut-off, the NPV was 99.5% and the PPV was 21.9%.

**Table 3:** Accuracy measures for FCR-1r with FCRI-SF score as reference standard

FCR-1r cut-off	Cases of FCRI-SF ≥ 22	Sensitivity	Specificity	PPV	NPV
≥ 1	92	1.00	0.309	0.079	1.000
≥ 2	92	1.00	0.474	0.101	1.000
≥ 3	92	1.00	0.624	0.135	1.000
≥ 4	89	0.967	0.746	0.183	0.997
≥ 5	86	0.935	0.804	0.219	0.995
≥ 6	69	0.750	0.897	0.300	0.984
≥ 7	57	0.620	0.928	0.337	0.976
≥ 8	44	0.478	0.954	0.379	0.969
≥ 9	19	0.207	0.978	0.358	0.954
≥ 10	16	0.174	0.991	0.533	0.953

Using the FCRI-SF cut-off of 16 or greater [20] indicating potential severe FCR, there were 349 positive cases. The AUC was 0.85 (95% CI 0.83–0.87, *p* < 0.0000) for the FCR-1r indicating good classification ability. Using the FCRI-SF cut-off of 13 or greater [20] as a rapid screening measure, there were 605 positive cases. The AUC was 0.85 (95% CI 0.83–0.86, *p* < 0.0000) for the FCR-1r indicating good classification ability. For ROC curves and accuracy measures of these cut-offs see Figs. 1 and 2 in supplementary material.

## Discussion

Valid psychosocial screening instruments with clinically relevant cut-offs are crucial to personalizing survivorship care. This study aimed to assess the validity of the FCR-1r and evaluate its screening performance in older versus younger CRC survivors with no recurrence of cancer. The FCR-1r demonstrated satisfactory concurrent, convergent, and divergent validity compared to the FCRI-SF and measures of constructs related to FCR. A FCR-1r cut-off score ≥ 5 provided an optimal balance of sensitivity, specificity, NPV, and PPV for identifying likely cases of clinically significant FCR (FCRI-SF ≥ 22) in practice. The validity of the FCR-1r was comparable to the original FCR-1 [12], and the classification ability in this population seems adequate.

While this study provides important evidence regarding the validity and screening performance of the FCR-1r in a population of cancer survivors with lower rates of clinical FCR compared to previous findings in CRC survivors [17, 35], further work is needed to confirm the unusually low prevalence of clinical FCR in older populations with different health care systems and to confirm the utility of the FCR-1r as a screening tool for FCR across different survivorship stages, especially in patients with recurrent disease.

Short screening questionnaires can advantageously be followed by longer and more detailed measurements like the FCRI-SF in a two-step screening process to confirm FCR severity, as recommended for depression [36]. In the current context, the high specificity is particularly reassuring, as only 20% of patients (i.e., those reporting false positives) would go through this step unnecessarily. The NPV is extremely high regardless of cut-off, due to the low number of cases in this cohort [37], but the low PPV underpins the need for a two-step approach.

Other globally used psycho-oncology screening measures like the Distress Thermometer (DT) and the ESAS assess fears and anxiety, but not specifically FCR and FCR might occur as an isolated focus of worry not captured by these. For example, high overall distress and a positive fear item on the problem list on the DT [38] showed low NPV and sensitivity to detect high FCR (AUC around 0.70 depending on cut-off on the Cancer Worry Scale (CWS-6)) [39], indicating that a high score could be due to high FCR, but could also reflect other causes of distress, and a low proportion of patients with high FCR would be identified using the DT. No subsequently detailed measurements can detect cases if they do not proceed to this next step, and the DT was deemed unsuitable for FCR screening in routine practice.

In contrast, the ESAS-*r* [22] anxiety item showed surprisingly good screening performance (AUC = 0.87) in detecting likely cases of clinical FCR in the FCR-1r validation study by Smith et al. [13], comparable to the FCR-1r (AUC = 0.89) and the FCR-1r in our study (AUC = 0.92). Not all patients might recognize FCR as fear, as this generic wording could also be interpreted more broadly as related to the impact of cancer on health, family, or work rather than recurrence in particular, arguably anxiety and FCR seems closer related.

If FCR-1r was expected to be a stand-alone question, it could be just as easy to ask the survivor about FCR orally at the medical encounter, but this rarely happens [40, 41]. Therefore, routine screening is recommended. FCR is only one of many other late effects present among long-term cancer survivors, such as general distress, cognitive impairment, sexual dysfunction, financial toxicity, and fatigue [42], and a larger battery of screening measures should be included in the psychosocial screening. The FCR-1r is valid, time-efficient, and simple to interpret. It can be easily integrated into existing screening procedures in routine survivorship care, and it gives a clear indication of whether it is relevant to spend time at the consultation discussing FCR and possible treatment options.

## Limitations

Missing data was an issue for the paper-and-pencil questionnaires and we also excluded more than 150 responses, with a response pattern of “0.” The fairly large number of responses excluded due to consistent responses despite the reversed item is not ideal. The strategy of imputation was to test two different methods and decide which one to proceed with, based on the impact on the datasets and on the analyses, and compared to complete case analyses. Multiple imputation might be a more powerful and elegant strategy, but besides being more comprehensive, the subsequent STATA prefix “mi estimate” limits the variety of analyses supported by STATA. Mean imputation is easily applicable in clinical and research perspectives and our results confirm the validity of this more accessible strategy, as no difference was observed between imputation styles on the interrelationship between FCRI-SF and FCR-1r. Mean imputation of the participant’s own mean can be used in future studies with missing FCRI-SF data.

As all CRC survivors in Denmark diagnosed between 2014 and 2018 were invited to participate in this study without indicating any sign of interest, the response rate was expected to be low. We do not have registry data on recurrence, but estimate, that about 25% of our population was not eligible for participation due to cancer recurrence. If these were excluded a priori we would reach a response rate of around 60%, consistent with comparable research in this field. Future studies could overcome this by seeking insight into medical records and only approach CRC survivors with no recurrence and no residual disease, or by including people living with or after recurrence of cancer.

Denmark is a highly digitalized country, and all strictly personal communication from the authorities is electronic unless you apply to receive messages by post. Digital literacy is closely, but not completely, related to age, and to comprehensively investigate the FCR level in all CRC survivors, we needed to distribute both paper and electronic surveys. Differences in respondents according to survey type were expected and corresponded to age and age-related characteristics like FCR level, employment status, marital status, education, and the clinical characteristics of cancer type and chemotherapy. Perhaps more importantly there were no differences in registry data between respondents and non-respondents, suggesting that our sample was representative. We have no reason to believe the severity of FCR is related to digital literacy, but this could be further investigated in future studies, especially as there are a growing number of online FCR interventions (e.g., iConquerFear).

Choosing another self-report measure as a gold standard is troublesome, as this measure too has limited accuracy for correctly classifying patients with clinical FCR. Investigating the capacity of the FCR-1r alone compared to a two-step model incorporating the FCR-1r against a gold-standard clinical interview would shed some light on which strategy is most suitable when considering burden on both health professionals and patients.

We focused on validating and testing the screening performance of a short FCR screening tool with high sensitivity and NPV for identifying clinically significant FCR in long-term CRC survivors with minimal patient burden, considering the relatively low prevalence of clinical FCR in this population. However, we note that the FCR-1r also demonstrated good classification ability in identifying less severe levels of FCR, which may be relevant to contexts where patient burden and resources available to provide intervention for lower FCR levels are less of a concern.

## Conclusion

Although the prevalence of patients reporting likely clinical FCR (FCRI-SF score ≥ 22) was low among long-term CRC survivors in our sample, particularly those who were older, the FCR-1r screening tool appears to be useful in identifying those who are likely to have clinical levels of FCR. The results suggest that using a cut-off score of ≥ 5 on the FCR-1r tool is appropriate for selecting survivors who may require further assessment and treatment.

### Supplementary Information

Below is the link to the electronic supplementary material.

Supplementary file1 (DOCX 222 KB)

## Data Availability

The dataset generated during this study is available from the corresponding author upon request.
