# Supplementary material for: Validity and screening capacity of the FCR-1r for fear of cancer recurrence in long-term colorectal cancer survivors
Source: Support Care Cancer. 2023 Nov 11;31(12):690. doi: 10.1007/s00520-023-08159-7 (PMC10638160; doi:10.1007/s00520-023-08159-7)

**SUPPLEMENTARY MATERIAL**

**Article title:** Validity and screening capacity of the FCR-1r for fear of cancer recurrence in long-term colorectal cancer survivors

**Journal name:** Supportive Care in Cancer

**Author names:** Johanne Dam Lyhne, Allan ‘Ben’ Smith, Signe Timm, Sébastien Simard, Lars Henrik Jensen, Lisbeth Frostholm, Per Fink

Corresponding author:
Dr Johanne Dam Lyhne
Department of Clinical Oncology
University Hospital of Southern Denmark
Beriderbakken 4, 7100 Vejle, Denmark
Phone: +45 2291 0740
Email: [Johanne.Dam.Lyhne@rsyd.dk](mailto:Johanne.Dam.Lyhne@rsyd.dk)

Table 1a. Demographic and clinical characteristics of the *paper* population, n=2,386

|  | Group 1 N=776 | Group 2 N=1,610 |
| --- | --- | --- |
| Age in years, mean, range (sd) | 81, 54 – 101 (7.3) | 80, 27 – 103 (8.3) |
| Gender, female | 405 (52%) | 966 (60%) |
| Cancer type  Colon  Rectum | 574 (74%)  202 (26%) | 1207 (75%)  403 (25%) |
| Metastatic disease  No  Yes  Missing | 740 (95%)  34 (4%)  2 (0%) | 1500 (93%)  85 (5%)  25 (2%) |
| WHO performance status, mean (sd) | 0.5 (0.7) | 0.6 (0.7) |

Group 1: CRCs willing and eligible to participate (n=575), including those with missings (201).
Group 2: Non-respondents (n=1,446) and CRCs excluded during data entering (n=164)

Table 1b. Demographic and clinical characteristics of the *e-Boks* population, n=2,097

|  | Group 1 N=1,124 | Group 2 N=973 |
| --- | --- | --- |
| Age in years, mean, range (sd) | 72, 38 – 94 (8.9) | 74, 29 – 98 (10.5) |
| Gender, female | 482 (43%) | 405 (42%) |
| Cancer type  Colon  Rectum | 742 (66%)  382 (34%) | 671 (69%)  302 (31%) |
| Metastatic disease  No  Yes  Missing | 1,059 (94%)  51 (5%)  14 (1%) | 909 (93%)  56 (6%)  8 (1%) |
| WHO performance status, mean (sd) | 0.2 (0.5) | 0.3 (0.5) |

Group 1: CRCs willing and eligible to participate (n=1,079), including those with missings (n=45)
Group 2: Non-respondents (n=909) and CRCs excluded during data entering (n=64)

Table 2. Demographic and clinical characteristics of paper-respondents and e-Boks-respondents. Numbers in parentheses are percentages unless otherwise specified.

|  | *Papers, N=575* | *E-Boks, N=1,079* |
| --- | --- | --- |
| Age in years, median, mean, range (sd) | **81**, 81, 54 - 98 (7.4) | **73,** 72, 38 – 94 (8.9) |
| Gender, female | 312 (54%) | 470 (44%) |
| Cancer type  Colon  Rectum | 422 (73%)  153 (27%) | 707 (65%)  382 (35%) |
| Mean FCR-1r score (sd) | 2.2 (2.5) | 2.8 (2.6) |
| Mean FCRI-SF score (sd) | 9.6 (6.8) | 10.4 (6.6) |
| Years since diagnose, mean, range (sd) | 5.4, 2.9 – 7.7 (1.4) | 6.7, 4.4 – 9.2 (1.3) |
| Time since last follow-up  > 1 year  3 – 12 months  1 week – 3 months  < 1 week  Missing | 380 (66%)  116 (20%)  44 (8%)  9 (2%)  26 (5%) | 734 (68%)  263 (24%)  74 (7%)  8 (1%)  0 (0%) |
| Time to next follow-up  < 1 week  1 weeks – 3 months  3 months – 12 months  > 1 year  Cancer control is ended  Missing | 3 (1%)  38 (7%)  51 (9%)  74 (13%)  368 (64%)  41 (7%) | 7 (1%)  71 (7%)  136 (13%)  295 (27%)  570 (53%)  0 (0%) |
| Chemotherapy received | 142 (24%) | 404 (37%) |
| Radiotherapy received | 45 (8%) | 80 (7%) |
| Metastatic disease at time of diagnosis  No  Yes  Missing | 545 (95%)  28 (5%)  2 (0%) | 1,020 (94%)  48 (4%)  11 (1%) |
| WHO performance status, mean (sd) | 0.4 (0.7) | 0.2 (0.5) |
| Marital status  Married  Not married  Divorced/separated  Widowed  Living together  Missing | 230 (40%)  52 (9%)  53 (9%)  216 (38%)  21 (4%)  3 (1 %) | 724 (67%)  78 (7%)  80 (7%)  134 (12%)  59 (5%)  4 (0%) |
| Employment status  Employed  Has been employed  Has never been employed  Missing | 22 (4%)  502 (87%)  37 (6%)  14 (2%) | 260 (24%)  793 (73%)  11 (1%)  15 (1%) |
| Education, any level, yes | 346 (60%) | 873 (80%) |
| Citizenship, Danish | 545 (95%) | 1,045 (97%) |
| Children, yes | 464 (81%) | 957 (89%) |

Table 3. Missing and imputed observations for each individual FCRI-SF items

**
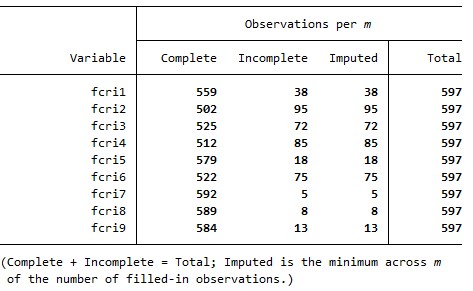
**

Table 4. Comparison of mean imputation and multiple imputation

| Data set: | Complete cases, n=434 | Mean imputation, n=575 | Multiple imputation, n=575 |
| --- | --- | --- | --- |
| Cases of FCRI-SF ≥ 22 | 27 | 31 | 30 |
| Regress fcr-1r fcriscore | 0.27 CI(0.25 – 0.29), r= 0.59 | 0.27 CI(0.25 – 0.29), r=0.52 | 0.28 (0.26 – 0.30) |

Table 5. Correlations between FCR-1r and individual FCRI-SF items (n=1,654).

|  | FCR-1r |
| --- | --- |
| FCRI-SF item 1 | 0.72, p<0.0001 |
| FCRI-SF item 2 | 0.68, p<0.0001 |
| FCRI-SF item 3 | 0.45, p<0.0001 |
| FCRI-SF item 4 | 0.52, p<0.0001 |
| FCRI-SF item 5 | 0.18, p<0.0001 |
| FCRI-SF item 6 | 0.56, p<0.0001 |
| FCRI-SF item 7 | 0.60, p<0.0001 |
| FCRI-SF item 8 | 0.51, p<0.0001 |
| FCRI-SF item 9 | 0.54, p<0.0001 |

Table 6. Wilcoxon rank-sum test of the difference between gender (n=1,654)

|  | FCR-1r Median (p25, p75) | *p* | FCRI-SF Median (p25, p75) | *p* |
| --- | --- | --- | --- | --- |
| Male Female | 2 (0, 4)  2 (0, 5) | *p* < 0.0001 | 8.2 (5, 14)  11 (5, 16) | *p* < 0.0001 |


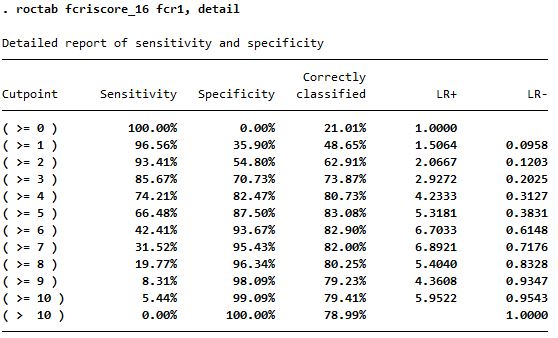

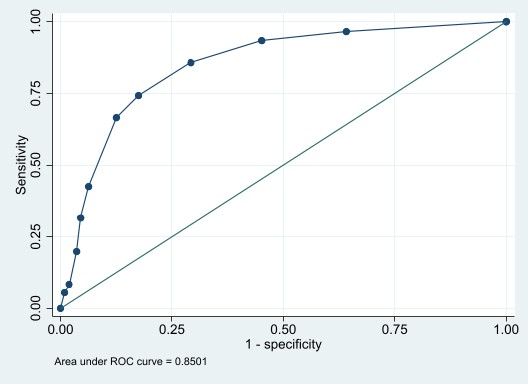
Figure 1. Receiver Operating Characteristic (ROC) curve evaluating the ability of the FCR-1r to discriminate between potential severe FCR and no potential severe FCR (Cut-off ≥ 16) and accuracy measures for the total sample.


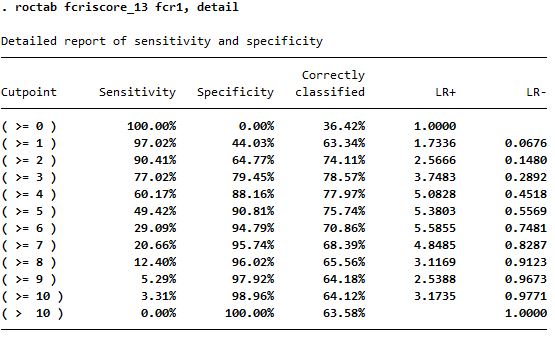
Figure 2. Receiver Operating Characteristic (ROC) curve evaluating the ability of the FCR-1r to discriminate between any level of FCR and no FCR (Cut-off ≥ 13) and accuracy measures for the total sample.


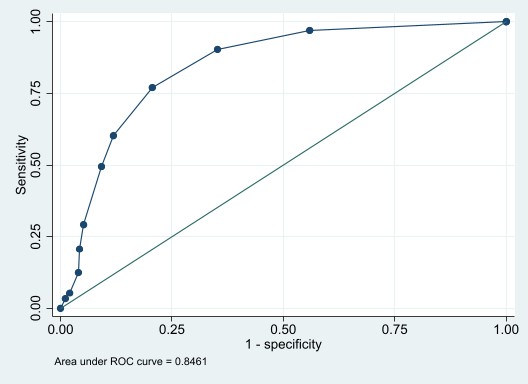

Supplement: Supplementary file 1 — Supplementary file1 (DOCX 222 KB) [file 520_2023_8159_MOESM1_ESM.docx]
